# Molecular characterization of *Pasteurella multocida* from cats and antibiotic sensitivity of the isolates

**DOI:** 10.1002/vms3.1424

**Published:** 2024-03-22

**Authors:** Ali Ziagham, Darioush Gharibi, Bahman Mosallanejad, Reza Avizeh

**Affiliations:** ^1^ Graduated of Veterinary Medicine Shahid Chamran University of Ahvaz Ahvaz Iran; ^2^ Department of Pathobiology Faculty of Veterinary Medicine Shahid Chamran University of Ahvaz Ahvaz Iran; ^3^ Department of Clinical Sciences Faculty of Veterinary Medicine Shahid Chamran University of Ahvaz Ahvaz Iran

**Keywords:** antimicrobial resistance, cat, capsular type, LPS type, *Pasteurella multocida*, virulence gene

## Abstract

**Background:**

Companion animals, including dogs and cats, are frequently identified as sources of *Pasteurella multocida*, a bacterium that can be transmitted to humans and cause infections.

**Objectives:**

This survey defines the prevalence, antibiotic sensitivity, capsular types, lipopolysaccharide (LPS) types and virulence factors of *P. multocida* isolated from cats.

**Methods:**

A total of 100 specimens from various cat breeds were collected. *P. multocida* was characterized using both biochemical tests and PCR. Genotypes of isolates were determined using capsular and LPS typing methods. Additionally, virulotyping was performed by detecting the presence of 12 virulence‐associated genes. Disk diffusion was used to determine the antibiotic sensitivity of the isolates.

**Results:**

The prevalence of *P. multocida* in cats was 29%. Among the isolates, the majority were capsular type A (96.5%) and type D (3.4%), with a predominant presence of type A. Twenty‐six of the isolates (89.66%) belonged to LPS genotype L6, whereas three isolates (10.3%) belonged to genotype L3. Among the 12 virulence genes examined, *sodC*, *oma87*, *ptfA*, *nanB* and *ompH* showed remarkable prevalence (100%). The *toxA* gene was detected in four isolates (13.8%). Variations were observed in other virulence genes. The *nanH* gene was present in 93.1% of the isolates, whereas the *pfhA* gene was detected in 58.6% of the isolates. The *exbD‐tonB*, *hgbB*, *sodA* and *hgbA* genes showed prevalence rates of 96.5%, 96.5%, 96.5% and 82.8%, respectively. Additionally, particular capsule and LPS types were associated with specific virulence genes. Specifically, the *toxA* and *pfhA* genes were found to be more prevalent in isolates with capsular type A and LPS genotype L6. Most isolates were resistant to ampicillin, clindamycin, lincomycin, streptomycin and penicillin.

**Conclusions:**

According to this epidemiological and molecular data, *P. multocida* from cats possess several virulence‐associated genes and are resistant to antimicrobial medicines commonly used in humans and animals. Thus, it is crucial to consider the public health concerns of *P. multocida* in humans.

## Graphical abstract

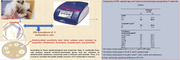

## 1 INTRODUCTION

As global economic and social development continues, there is a rapid growth in human material and spiritual needs. This growth is expected to lead to an increased interest in keeping and breeding various animals, including cats, dogs and food‐producing animals such as pigs and cattle. This increases the risk of zoonosis transmission to humans. The family of *Pasteurellaceae* includes commensals and potential pathogens of birds and mammals. These pathogens are significant for human health and animal production. Most pathogenic members of *Pasteurellaceae* reside on the mucus membranes of the respiratory, oral and genital tracts, in various species, including dogs, cats, ruminants and pigs. *Pasteurella multocida* is a gram‐negative bacterium commonly found in the upper respiratory tract of many animal species. Transmission of *P. multocida* occurs through inhalation, scratches, bites or contact with carriers or infected animals (Boyce et al., 2012). It has been documented that *P. multocida* has been isolated from approximately 50% of dog bite wounds, 75% of cat bite wounds or scratches, and, less frequently, from licks (Talan et al., 1999). *P. multocida* can cause various economically significant animal infections as well as local and systemic wound infections (Wilson & Ho, 2013). Peng et al. reported 330 human infections caused by *P. multocida* between 1975 and 2022, primarily resulting from dogs and cats biting, scratching or licking (Peng et al., 2022). It has been acknowledged that over 60% of U.S. households have at least one pet (Applebaum et al., 2023). The United States sees approximately 300,000 (1%) emergency room visits annually due to animal bites and scratches (Wilson & Ho, 2013). Although not all of these lead to clinically relevant infections, *Pasteurella* species are isolated from 50% to 75% of dog and cat bites, respectively (Wilson & Ho, 2013). Approximately 8 million cats and dogs are kept as pets in Iran (Yusefi et al., 2019). However, no studies have been conducted on *P. multocida* in cats or on human infection cases associated with cats. Human infection with *P. multocida* is inevitable, particularly for those in contact with animals, especially companion animals (dogs and cats). Therefore, *P. multocida* might pose a health risk for humans in the future; hence, the public health authorities should not ignore it.

In cats, *P. multocida* causes rhinitis, conjunctivitis, pneumonia, gingivitis, stomatitis, abscesses and sometimes osteonecrosis (Ferreira et al., 2015). A cat bite infection with *P. multocida* can lead to lymphangitis, cellulitis, peritonitis, abscesses and septic arthritis in humans (Abreu et al., 2018). In immunocompromised patients, *P. multocida* can cause soft tissue infections, peritonitis, pneumonia, urinary tract infections, bacteraemia, septic shock, meningitis and even death (Elad, 2013; Kofteridis et al., 2009; Larnè et al., 2018; Sol et al., 2013).

*P. multocida* produces many virulence factors, such as lipopolysaccharide (LPS), PMT (*P. multocida* toxin), capsules and adhesins. According to their capsular polysaccharide antigens, isolates can be differentiated serologically into serogroups A, B, D, E and F (Chung et al., 1998). The pathogenicity of *P. multocida* depends on outer membrane proteins (OMPs), capsular antigens and virulence genes, such as dermonecrotoxin (*toxA*), fimbriae, adherence and colonization factors (*pfhA*, *ptfA*), iron‐regulated and acquisition proteins (*exbD*‐*tonB*, *hgbA* and *hgbB*) and extracellular enzymes, such as neuraminidase (*nanB* and *nanH*), superoxide dismutase (*sodA* and *sodC*) and OMPs (*ompA*, *ompH* and *oma87*) (Bosch et al., 2002; Cox et al., 2003; Ewers et al., 2006, Ewers et al., 2006, Harper & Boyce, 2017). Antibodies that target virulence factors such as LPS are considered one of the most effective ways of protecting against *P. multocida*. Vaccines need to be developed that target the types of LPS expressed by various *P. multocida* strains, including cat isolates (Harper & Boyce, 2017).

Administration of prolonged and indiscriminate antibiotics has led to antibiotic resistance and the development of multidrug‐resistant strains in *P. multocida* (Arora et al., 2005). Antimicrobial resistance in *P. multocida* can vary depending on the host species, time and geographical origin (Shivachandra et al., 2004). The frequency of virulence factors and antimicrobial sensitivity of *P. multocida* in cats has not been extensively investigated. This lack of understanding makes it challenging to develop effective treatment strategies and prevent infections caused by this bacteria. This study aimed to investigate the prevalence, capsular and LPS types, virulence genes and antibiotic sensitivity of *P. multocida* isolated from cats’ oral cavities.

## 2 MATERIALS AND METHODS

### 2.1 Sampling

A total of 100 specimens were collected from apparently healthy owned cats which had not taken antibiotics or other medications in the past 3 months and had no history of respiratory or gastrointestinal illnesses.

The samples were collected using sterile swabs from the oral cavity of different cat breeds, including DSH (65 cats), Persian (28 cats), Scottish (6 cats) and Tibetan (1 cat). The examination included 53 male and 47 female cats, ranging in age from 2 months to 10 years. Among the cats, 29 were younger than 1 year, 53 were between 1 and 3 years old and 18 were older than 3 years old.

### 2.2 Bacterial isolation, identification and confirmation

The collected specimens were plated on 10% sheep blood agar and incubated aerobically at 37°C overnight. Colonies with small, mucoid and convex appearances were selected for further biochemical analysis. Microscopic examination was performed to observe the morphology and gram stain structure of the isolates. Biochemical tests, including catalase, oxidase, indole, Simmons' citrate, methyl red, Voges–Proskauer, nitrate reduction and growth on MacConkey, were performed following the instructions provided by Clinical Veterinary Microbiology (Markey et al., 2013).

To confirm the presence of *P. multocida*, molecular analysis was conducted. A bacterial colony suspected to be *P. multocida* was selected and added to 100 µL of sterile distilled water to prepare template DNA. The bacterial suspension was then subjected to a boiling water bath for 10 min to lyse the bacterial cells. Afterwards, the suspension was centrifuged at 4000 *g* for 4 min to collect the supernatant. The supernatant, containing the DNA sample, was stored at −20°C for further analysis. To specifically detect *P. multocida*, a specific PCR targeting a particular fragment of the *kmt1* gene was performed. The primers used for amplification were KMT1SP6 and KMT1T7, as described by Townsend et al. (2001) (Table 1). For the PCR reaction, a positive control strain of *P. multocida* (ATCC 43137) was used. The reaction mixture consisted of 25 µL, which included 7.5 µL of sterile ultrapure Milli‐Q water, 12.5 µL of Ampliqon 2× master mix (containing MgCl_2_ at a concentration of 1.5 mM), 0.5 µL of KMT1T7 primer (at a concentration of 10 mM) from Bioneer, 0.5 µL of KMT1SP6 primer (at a concentration of 10 mM) from Bioneer, and 4 µL of the DNA sample.

**TABLE 1:** Primers used for identification of *Pasteurella multocida* determine of capsular types, lipopolysaccharide (LPS) types and virulence‐associated genes in *Pasteurella* strains.

Description tested features	Gene	Primers	Sequences (5'–3')	References
*P. multocida*	*kmt1*	KMT1T7 KMT1SP6	ATCCGCTATTTACCCAGTGG GCTGTAAACGAACTCGCCAC	Townsend et al. (1998)
Serogroup A	*hyaD‐hyaC*	CAPA‐FWD CAPA‐REV	TGCCAAAATCGCAGTCAG TTGCCATCATTGTCAGTG	Townsend et al. (2001)
Serogroup B	*bcbD*	CAPB‐FWD CAPB‐REV	GCCCGAGAGTTTCAATCC CATTTATCCAAGCTCCACC	
Serogroup D	*dcbF*	CAPD‐FWD CAPD‐REV	TTACAAAAGAAAGACTAGGAGCCC CATCTACCCACTCAACCATATCAG	
Serogroup E	*ecbJ*	CAPE‐FWD CAPE‐REV	TCCGCAGAAAATTATTGACTC GCTTGCTGCTTGATTTTGTC	
Serogroup F	*fcbD*	CAPF‐FWD CAPF‐REV	AATCGGAGAACGCAGAAATCAG TTCCGCCGTCAATTACTCTG	
LPS type 1	*pcgD* *pcgB*	BAP6119‐FWD BAP6120‐ REV	ACATTCCAGATAATACACCCG ATTGGAGCACCTAGTAACCC	Harper et al. (2015)
LPS type 2	*nctA*	BAP6121‐ FWD BAP6122‐ REV	CTTAAAGTAACACTCGCTATTGC TTTGATTTCCCTTGGGATAGC	
LPS type 3	*gatF*	BAP7213‐FWD BAP7214‐ REV	TGCAGGCGAGAGTTGATAAACCATC CAAAGATTGGTTCCAAATCTGAATGGA	
LPS type 4	*latB*	BAP6125‐ FWD BAP6126‐ REV	TTTCCATAGATTAGCAATGCCG CTTTATTTGGTCTTTATATATACC	
LPS type 5	*rmlA* *rmlC*	BAP6129‐ FWD BAP6130‐ REV	AGATTGCATGGCGAAATGGC CAATCCTCGTAAGACCCCC	
LPS type 6	*nctB*	BAP7292‐ FWD BAP7293‐ REV	TCTTTATAATTATACTCTCCCAAGG AATGAAGGTTTAAAAGAGATAGCTGGAG	
LPS type 7	*ppgB*	BAP6127‐ FWD BAP6128‐ REV	CCTATATTTATATCTCCTCCCC CTAATATATAAACCATCCAACGC	
LPS type 8	*natG*	BAP6133‐ FWD BAP6134‐ REV	GAGAGTTACAAAAATGATCGGC TCCTGGTTCATATATAGGTAGG	
Dermonecrotoxin	*toxA*	TOXA‐FWD TOXA‐REV	TCTTAGATGAGCGACAAGG GAATGCCACACCTCTATAG	Lichtensteiger et al. (1996)
Outer membrane protein involved in iron acquisition	*hgbA*	HGBA‐FWD HGBA‐REV	TGGCGGATAGTCATCAAG CCAAAGAACCACTACCCA	Ewers et al. (2006)
Outer membrane protein involved in iron acquisition	*hgbB*	HGBB‐FWD HGBB‐REV	TCATTGAGTACGGCTTGAC CTTACGTCAGTAACACTCG	Atashpaz et al. (2009)
Filamentous haemagglutinin adhesin	*pfhA*	PFHA‐FWD PFHA‐REV	AGCTGATCAAGTGGTGAAC TGGTACATTGGTGAATGCTG	Ewers et al. (2006)
Porin	*oompH*	OMPH‐FWD OMPH‐REV	CGCGTATGAAGGTTTAGGT TTTAGATTGTGCGTAGTCAAC	Ewers et al. (2006)
Porin	*oma87*	OMA87‐FWD OMA87‐REV	ATGAAAAAACTTTTAATTGCGAGC TGACTTGCGCAGTTGCATAAC	Ewers et al. (2006)
Superoxide dismutase	*soda*	SODA‐FWD SODA‐REV	TACCAGAATTAGGCTACGC GAAACGGGTTGCTGCCGCT	Ewers et al. (2006)
Superoxide dismutase	*sodC*	SODC‐FWD SODC‐REV	AGTTAGTAGCGGGGTTGGCA TGGTGCTGGGTGATCATCATG	Lainson et al. (1996)
Iron metabolism	ex*BD*‐*tonB*	TONB‐FWD TONB‐REV	GGTGGTGATATTGATGCGGC GCATCATGCGTGCACGGTT	Ewers et al. (2006)
Sialidase	*nanB*	NANB‐FWD NANB‐REV	GTCCTATAAAGTGACGCCGA ACAGCAAAGGAAGACTGTCC	Ewers et al. (2006)
Sialidase	*nanH*	NANH‐FWD NANH‐REV	GAATATTTGGGCGGCAACA TTCTCGCCCTGTCATCACT	Ewers et al. (2006)
Type IV fimbriae	*ptfA*	PTFA‐FWD PTFA‐REV	TGTGGAATTCAGCATTTTAGTGTGTC TCATGAATTCTTATGCGCAAAATCCTGCTGG	Doughty et al. (2000)

### 2.3 Capsular typing by multiplex PCR

To determine the capsular types of the isolates, genotyping was performed using specific primers for capsular types capA, capB, capD, capE and capF, as described by Townsend et al. (2001). The PCR reaction mixture contained a DNA template, 2× Ampliqon PCR master mix from Ampliqon, sterile ultrapure Milli‐Q water and a set of primers from Bioneer. The final concentration of each primer in the reaction mixture was 3.2 mM. Table 1 shows the primer sequences. The amplification was based on the method described by Townsend et al. (2001), with slight modifications. As positive controls for each capsular type, the reference strains of *P. multocida* ATCC 43137 and HN06 were used for capsular types A (CapA) and D (CapD), respectively.

### 2.4 LPS typing by multiplex PCR

The isolates were genotyped following the method described by Harper et al. (2015). This involved using specific primers for the LPS outer core biosynthesis genes, including Pcg, nctA, gatF, latB, nctB, rmlA, ppgB and natG. The primer sequences can be found in Table 1. The amplification process was conducted following the protocol outlined by Harper et al. (2015) with slight modifications. The PCR reactions were carried out in a final volume of 50 µL, with the following reagent concentrations: Ampliqon 2× master mix (containing MgCl_2_ at a concentration of 1.5 mM), sterile ultrapure Milli‐Q water and a set of eight primer pairs at a final concentration of 3.2 mM for each primer pair. Additionally, 3 µL of template DNA was added to the reaction mixture. To serve as positive controls for the LPS types, reference strains of *P. multocida* ATCC 43137 and ATCC 2100 were utilized for LPS types L3 and L6, respectively.

### 2.5 Virulence‐associated genes detection

The detection of virulence genes was performed using three multiplex‐PCR procedures following the protocols described by Atashpaz et al. (2009), Ewers et al. (2006) and Lichtensteiger et al. (1996) with minor modifications. The primer sequences can be found in Table 1. For each amplification reaction, a reagent mixture (25 µL) was prepared at the following composition and concentrations; Ampliqon 2× master mix (containing MgCl_2_ at a concentration of 1.5 mM), sterile ultrapure Milli‐Q water and the set of four primer pairs at a final concentration of 0.5 µL (10 picomols) from each primer pairs. Additionally, 5 µL of template DNA was added to the reaction mixture. The reference strains for each virulence gene of *P. multocida*, were ATCC 12945 (*pfhA*, *sodA*, *hgbA* and *ptfA*), ATCC 43137 (*oma87*, *ptfA, nanB* and *nanH*) and HN06 (*hgbB*).

### 2.6 PCR and electrophoresis

PCR reactions were conducted using an Eppendorf MasterCycler Thermal Cycler. *Mannheimia haemolytica* ATCC 29694 was used as a negative control for all PCR tests. The amplified products were separated by electrophoresis on a 1.5% agarose gel, stained with safe DNA gel stain, visualized using safe UV equipment, and imaged.

### 2.7 Antimicrobial sensitivity testing

Antimicrobial sensitivity testing was performed using a disc diffusion test, following the recommendations of the Clinical and Laboratory Standards Institute (Andrews et al., 2004, CLSI, 2017, CLSI, 2015, Citron et al., 2005). The antimicrobial discs (PadtanTeb Co) used included trimethoprim–sulfamethoxazole (23/7 µg), nitrofurantoin (300 µg), fluorophenicol (30 µg), doxycycline (30 µg), ceftriaxone (30 µg), co‐amoxiclav (20/10 µg), cefazolin (30 µg), tetracycline (30 µg), ampicillin (10 µg), clindamycin (2 µg), lincomycin (2 µg), streptomycin (10 µg) and penicillin (10 µg).

### 2.8 Data analysis

The analysis of the results was conducted using SPSS (version 23), with statistical significance set at a *p*‐value less than 0.05. Chi‐square and Fisher's exact tests were used to analyse gene combinations among *P. multocida* isolates.

## 3 RESULTS

### 3.1 Prevalence of *P. multocida*

**FIGURE 1:**
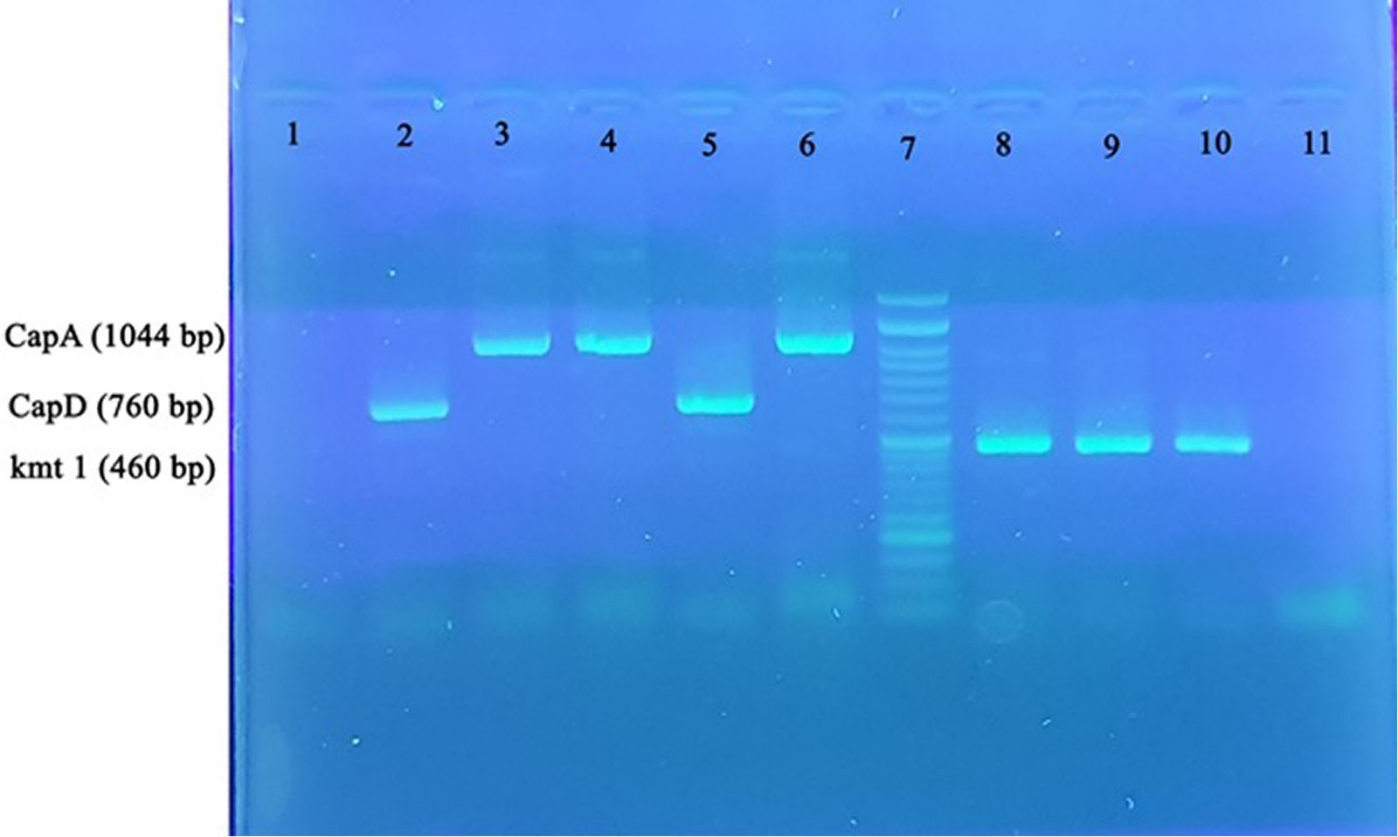
PCR profiles of amplifications of *kmt1* gene and capsule genes (A and D). The upper panel shows the following: Lane 1: capsule negative control; Lane 2: capsule type D positive control (HN06); Lane 3: capsule type A positive control (ATCC 43137); Lanes 4 and 6: *Pasteurella multocida* capsular type A; Lane 5: *P. multocida* capsular type D; Lane 7: 50 bp DNA marker; Lanes 8 and 9: *P. multocida*; Lane 10: *kmt1* gene positive control; Lane 11: *kmt1* gene negative control.

The biochemical tests conducted on bacterial isolates obtained from 100 cats revealed that 39% of them carried *Pasteurella* species. Using PCR, a DNA band of approximately 460 bp confirmed the identity of 29% of the isolates as *P. multocida*, indicating its widespread presence in cats (Figure 1). The prevalence of *P. multocida* varied among different age groups, with 13.8% of cats less than a year old, 37.7% of those between 1 and 3 years old and 27.7% of those older than 3 years old being infected. The prevalence was 28.3% in male cats and 29.8% in female cats. Among different cat breeds, the prevalence of *P. multocida* was 27.7% in DSH, 28.6% in Persian, 50% in Scottish and 0% in Tibetan cats. Statistical analysis revealed that there was no significant relationship among the age, gender, breed of cats and the infection with *P. multocida* (*p* < 0.05).

### 3.2 Capsular typing by multiplex PCR

The amplified DNA products of approximately 1044 and 657 bp (Figure 1) were identified as *P. multocida* CapA and CapD, respectively. Capsular genotyping of the *P. multocida* isolates revealed two capsular types: A (28 isolates) and D (1 isolate), with prevalence rates of 96.5% and 3.4%, respectively. Capsular types B, E and F were not detected among the isolates. The dominant capsular type among the cat isolates was type A.

### 3.3 LPS genotyping using PCR analyses

**FIGURE 2:**
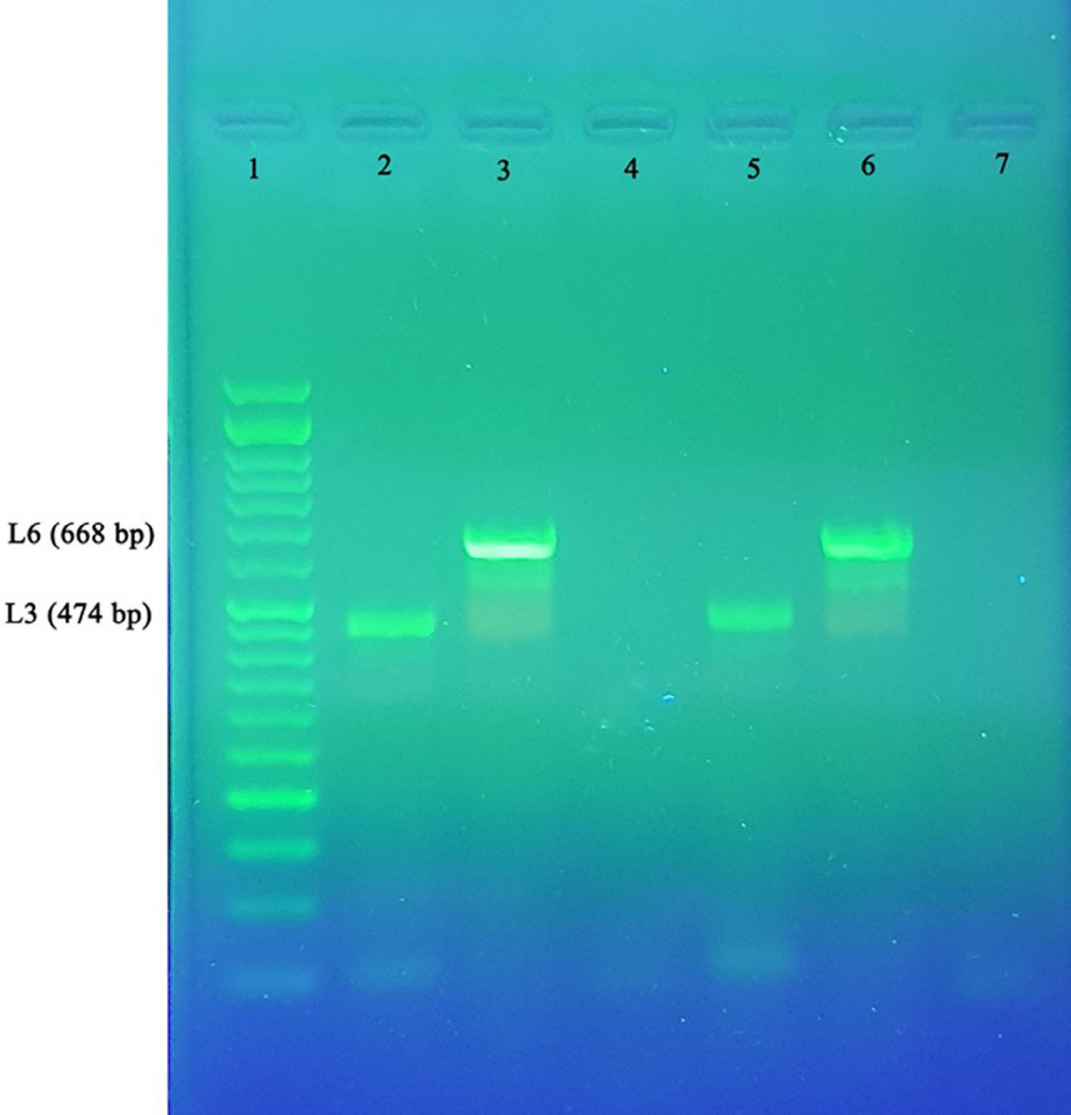
PCR product of lipopolysaccharide (LPS) types 3 and 6 on 1.5% agarose gel; Lane 1: 50 bp ladder marker; Lane 2: L3 positive control (ATCC 43137); Lane 3: L6 positive control (HN06); Lane 4 and 7: negative control; Lanes 5 and 6: samples.

The LPS genotyping of the isolates was performed using eight specific primers targeting the LPS outer core biosynthesis loci. Among the 29 isolates of *P. multocida*, 26 (89.66%) were identified as genotype L6, whereas 3 (10.3%) were genotype L3 (Figure 2).

### 3.4 Virulence genes detection using PCR analyses

**FIGURE 3:**
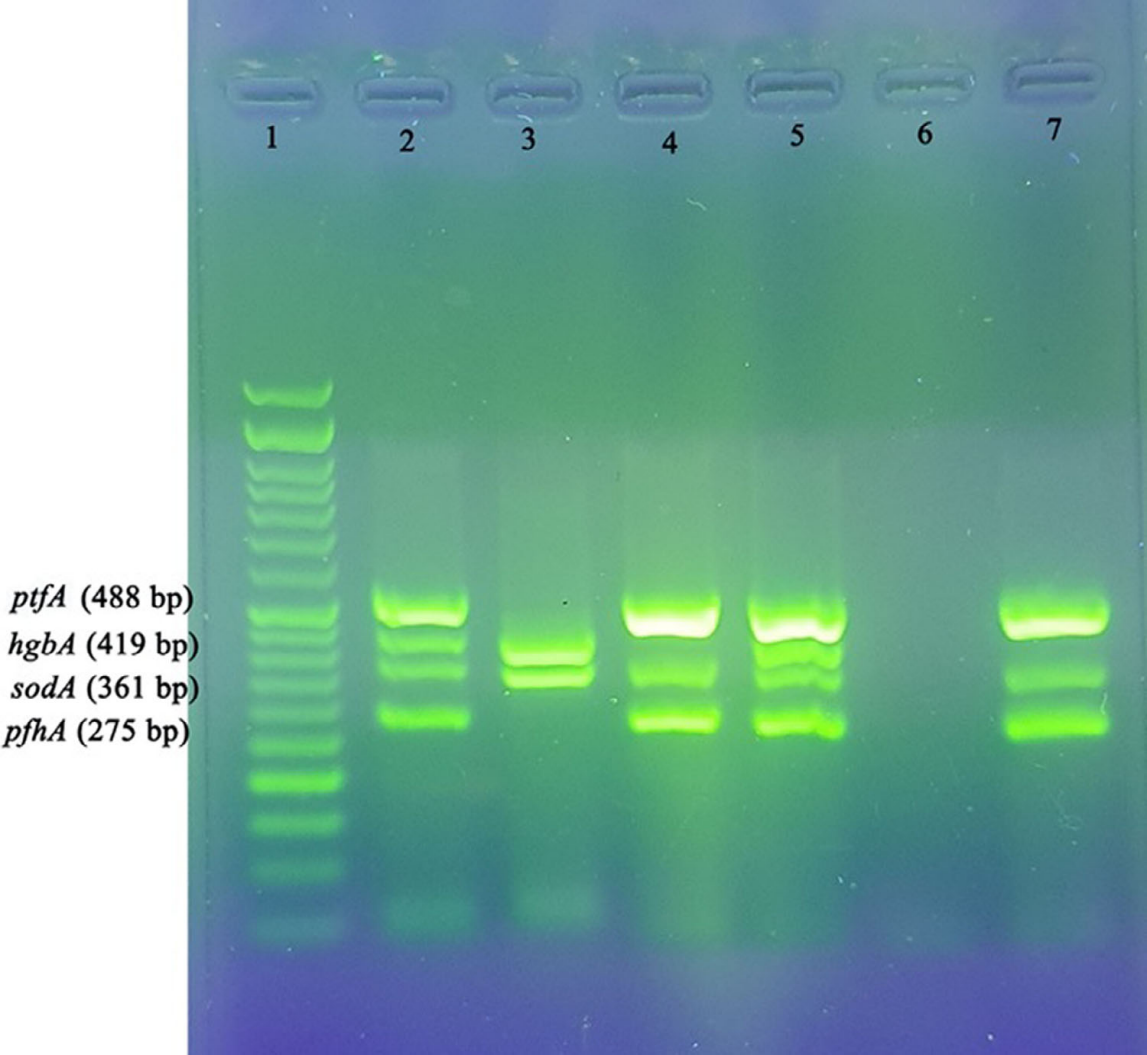
Virulence gene detection using multiplex PCR. Lane 1: 50 bp DNA marker; Lane 2: *Pasteurella multocida* ATCC 12945; Lanes 3–5, 7: samples; Lane 6: negative control. Samples were electrophoresed at 90 V/cm for 45 min on a 1.5% agarose gel (1 × TAE) stained with Safe Stain, visualized by UV illumination, and photographed.

**FIGURE 4:**
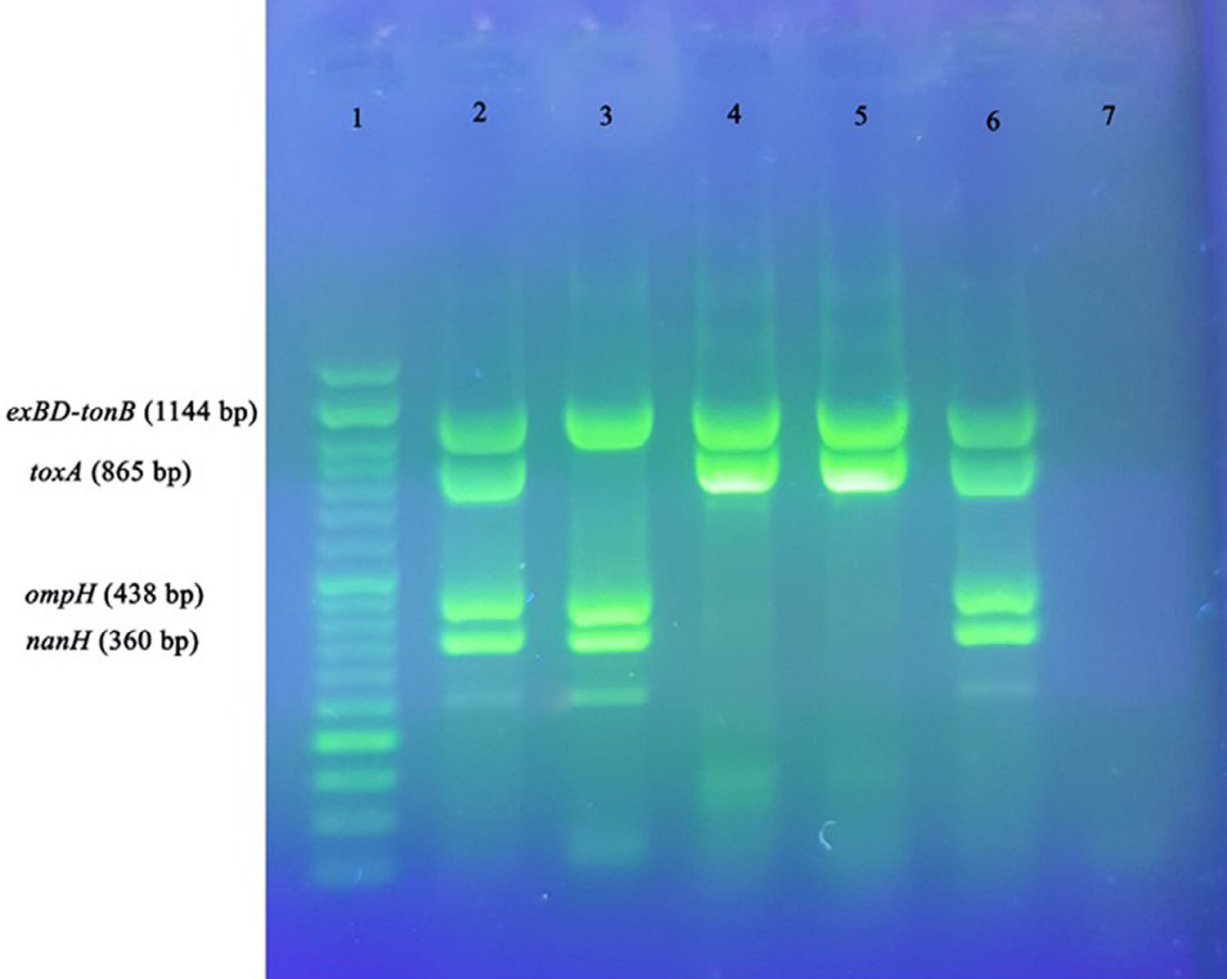
Virulence gene detection using multiplex PCR. Lane 1: 50 bp DNA marker; Lane 2: positive control; Lanes 3–6: samples; Lane 7: negative control. Samples were electrophoresed at 90 V/cm for 45 min on a 1.5% agarose gel (1 × TAE) stained with Safe Stain, visualized by UV illumination, and photographed.

**FIGURE 5:**
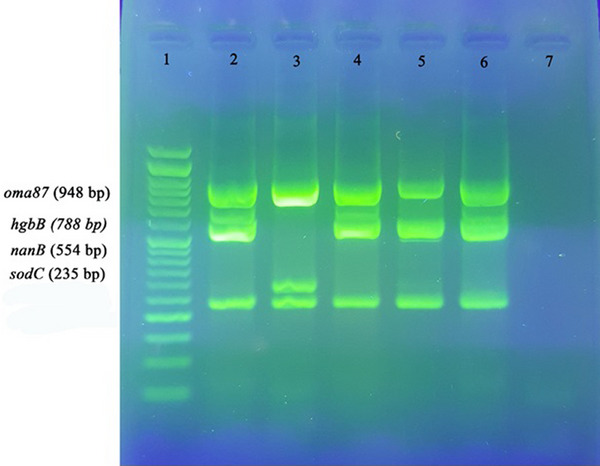
Virulence gene detection using multiplex PCR. Lane 1: 50 bp DNA marker; Lane 2: positive control; Lanes 3–6: samples; Lane 7: negative control. Samples were electrophoresed at 90 V/cm for 45 min on a 1.5% agarose gel (1 × TAE) stained with Safe Stain, visualized by UV illumination, and photographed.

A total of 12 virulence genes were examined in *P. multocida*, as shown in Figure 3 (*ptfA*, *pfhA*, *sodA* and *hgbA*), *exbD*‐*tonB*, *toxA*, *ompH* and *nanH* (Figure 4), and *oma87*, *hgbB*, *nanB* and *sodC* (Figure 5). Among these genes, *sodC*, *oma87*, *ompH*, *ptfA* and *nanB* were found to be present in all isolates (100%). The genes *hgbB*, *exbD*‐*tonB* and *sodA* were found in 96.5% of the isolates. Variations were observed in the presence of other virulence genes, with *nanH* being present in 93.1% of isolates, *hgbA* in 82.8% and *pfhA* in 58.6%. The *toxA* gene was detected in only 4 isolates (13.8%).

Furthermore, specific capsule and LPS types were associated with certain virulence genes. The *toxA* and *pfhA* genes were more prevalent in isolates with CapA and LPS type 6. It was also observed that the *toxA* and *pfhA* genes were detected exclusively in strains with LPS type 6 (*p* < 0.05). The results of the PCR analysis for the presence of different capsular types, LPS types and virulence genes are summarized in Table 2.

**TABLE 2:** Frequencies of lipopolysaccharide (LPS), capsular type and virulence‐associated genes among feline *Pasteurella multocida* isolates.

	Isolates *n* = 29	
	N	%
**LPS** ^a^		
L3	3	10.3
L6	**26**	**89.6**
**Capsular type** ^b^		
A	**28**	**96.5**
D	1	3.4
**Virulence associated genes** ^c^		
*exbD‐tonB*	28	96.5
*hgbB*	28	96.5
*Soda*	28	96.5
*nanH*	27	93.1
*hgbA*	24	82.8
*pfhA*	17	58.6
*toxA*	4	13.8

^Note:^ In bold, statistically significant differences (*p* < 0.05).

^a^ All isolates tested negative for 1, 2, 4, 5, 7 and 8 LPS types.

^b^ All isolates tested negative for B, E and F capsular types.

^c^ All isolates tested positive for *ptfA*, *oma87*, *sodC*, *ompH* and *nanB* genes.

### 3.5 Antimicrobial sensitivity testing

The susceptibility testing results showed that all isolates were susceptible to trimethoprim–sulfamethoxazole and nitrofurantoin. The isolates exhibited the highest susceptibility to fluorophenicol (97.05%), doxycycline (97.05%), ceftriaxone (97.05%), co‐amoxiclav (94.11%), cefazolin (94.11%) and tetracycline (91.17%), respectively.

On the other hand, the highest antibiotic resistance was observed for ampicillin (97.05%), followed by clindamycin (76.47%), lincomycin (67.64%), streptomycin (64.64%) and penicillin (58.82%).

## 4 DISCUSSION

Despite the importance of cats that are contaminated with *P. multocida*, which may transmit it to humans through bites and scratches, comprehensive information was not available regarding the distribution and prevalence of capsular and LPS types among regions. Hence, the current study aims to describe the prevalence, capsular and LPS genotyping, virulence factor profile and antibiotic sensitivity of feline *P. multocida* in Khuzestan province (southwestern Iran). Based on this survey, *P. multocida* is found to be prevalent in cats. Its reported frequency ranges from 10% to 90% (Giordano et al., 2015; Moyaert et al., 2017). Regional differences or the presence of competitive oral flora could account for these variations (Aski & Tabatabaei, 2016; Ferreira et al., 2015; Freshwater 2008; Moyaert et al., 2017). Cats have the highest carriage rates, ranging from 70% to 90%. The second most common carrier is dogs, with a rate of 20%–50% (Giordano et al., 2015). Consequently, one study found that *Pasteurella* species can be isolated in wound cultures in 50% of injuries caused by dog bites and 75% of injuries caused by cat bites (Talan et al., 1999). Due to the high frequency of *P. multocida* in cats and exposure to companion animals, these trends are thought to contribute to the increased risk of *P. multocida* transmission to humans. Our interactions with pets are unlikely to diminish in the future. Considering the high prevalence of *P. multocida* as part of the microbiota of domestic and wild animals, it would be prudent to consider the zoonotic transmission of *P. multocida* as a severe risk of infection.

Various virulence factors have been associated with the pathogenicity of *P. multocida*, including capsules, adhesins, toxins, siderophores, sialidases and OMPs. These virulence factors not only enhance colonization and invasion but also hinder or disrupt host defence mechanisms, cause tissue damage or inflammation, and provoke an inflammatory response in the host (Tang et al., 2009). This study revealed that the predominant capsular type in the isolates was type A (96.5%), followed by type D (3.4%). The prevalence of capsular type A in cat isolates aligns with previous studies (Arumugam et al., 2011; Aski & Tabatabaei, 2016; Ferreira et al., 2015). Moreover, in studies conducted by Prabhakar et al. (2012), Ewers et al. (2006), Mombeni et al. (2021), Kumar et al. (2004) and Aski and Tabatabaei (2016), it was reported that CapA is more commonly found than CapD in mammal isolates. Additionally, Wilson and Ho (2013) found that serogroups A and D are responsible for the majority of human infections. Capsule polysaccharides, which bear structural resemblance to vertebrate glycosaminoglycans, play a crucial role in molecular mimicry, immune evasion and resistance to phagocytosis (Harper & Boyce, 2017). In our study, we observed that feline isolates were classified into L3 and L6 LPS types, with L6 being the predominant type (26 out of 29 isolates, accounting for 89.66%), whereas L3 was less prevalent (3 out of 29 isolates). Due to a lack of knowledge regarding LPS types in previous studies, no correlation was identified between capsular and LPS types in cats. This study represents the first report on the prevalence of LPS types in *P. multocida* strains isolated from cats. Our findings revealed a correlation between CapA and L6. LPS is recognized as a key target for protective antibodies against *P. multocida*. To develop effective and cross‐protective vaccines, it is crucial to understand the LPS types expressed by *P. multocida* strains, including those isolated from cats (Harper & Boyce, 2017). The present study has contributed valuable epidemiological data regarding capsular and LPS types, virulence‐associated genes and antibiotic resistance among cat strains in Iran. The isolates showed a 100% prevalence of *ptfA*, *oma87*, *ompH*, *nanB* and *sodC*. However, variations were observed in other virulence genes, including *sodA* (96.5%), *toxA* (13.8%), *pfhA* (58.6%), *exbD‐tonB* (96.5%), *hgbA* (82.8%), *hgbB* (96.5%) and *nanH* (93.1%). In contrast, Ferreira et al. (2015) reported that none of the *P. multocida* strains in their study possessed the *toxA*, *tbpA* or *pfhA* genes. The high prevalence of certain genes, such as *pfhA* and *ptfA*, is indeed significant in terms of bacterial pathogenesis. These genes contribute to early bacterial colonization and survival in the oral cavity of cats, increasing the likelihood of human colonization if these strains are transmitted from cats to humans. In *P. multocida*, genes like *oma87* and *ompH* play a role in invasion and provide protection against toxic molecules. Additionally, these proteins have various functions in bacteria, including nutrient absorption, molecule import and export and close interaction with the host. The high prevalence of iron‐regulated and acquisition proteins (*exbD‐tonB*, *hgbA* and *hgbB*), extracellular enzymes such as neuraminidase (*nanB* and *nanH*) and superoxide dismutase (*sodA* and *sodC*) in *P. multocida* may contribute to enhanced pathogenicity in cats and humans, providing the bacterium with an added advantage. The study findings also indicated a noteworthy association between virulence genes and a specific LPS type. Specifically, the presence of *pfhA* and *toxA* genes was observed exclusively in isolates with LPS type 6 (L6). However, there is limited information available regarding the virulence genes and LPS types of *P. multocida* in cats. This information could be valuable in gaining a deeper understanding of the pathogenesis of *P. multocida* infections in cats and the potential transmission of infections to humans. Indeed, evaluating the virulence factors, LPS types and serogroups of *P. multocida* is crucial as it allows for the prediction of the organism's pathogenic behaviour. Additionally, capsular, LPS and virulotyping are valuable tools in genotyping *P. multocida* and serve as the foundation for designing region‐specific vaccines for future use. These approaches aid in understanding the pathogenicity of *P. multocida* and contribute to the development of effective preventive measures.

*P. multocida* has the ability to carry plasmids that provide resistance to various antibiotics, including beta‐lactams, tetracycline, streptomycin and sulphonamides (Kadlec et al., 2011). To ensure effective treatment of respiratory diseases and minimize economic losses, it is crucial to monitor the resistance of *P. multocida* in food‐producing animals. This helps in determining the appropriate first‐line treatment options. Given that *Pasteurella* is the most frequently isolated bacteria from dog and cat bites, it is important to conduct surveillance for human bites as well. Wound infections caused by *P. multocida* are commonly treated with broad‐spectrum antimicrobials. *P. multocida* isolates generally exhibit susceptibility to β‐lactam antibiotics. Additionally, second and third‐generation cephalosporins, tetracyclines, co‐trimoxazole and fluoroquinolones can be considered alternative treatment options, as they have shown positive activity against *P. multocida* (Kehrenberg et al., 2001; Larnè et al., 2018). *P. multocida* strains can exhibit variability in antibiotic resistance based on their geographical origin and prior exposure to antimicrobial treatments. It is important to consider these factors when selecting appropriate antibiotics for the treatment of *P. multocida* infections, as resistance patterns may differ between regions and individual cases. Regular surveillance and monitoring of antibiotic resistance in *P. multocida* strains can help guide treatment decisions and ensure effective management of infections. According to the study mentioned, trimethoprim–sulfamethoxazole, nitrofurantoin, fluorophenicol, doxycycline, ceftriaxone, co‐amoxiclav, cefazolin and tetracycline have been identified as the most effective therapeutic options against feline isolates of *P. multocida*. These antibiotics have shown high efficacy rates ranging from 91.17% to 100%. These findings are consistent with previous studies (Ferreira et al., 2012; Güler et al., 2013; Kehrenberg et al., 2001; Rigobelo et al., 2013; Yoshimura et al., 2001). Although in this study, all *P. multocida* isolates were sensitive to trimethoprim–sulfamethoxazole (100%), Tang et al. (2009) reported resistance to trimethoprim–sulfamethoxazole and Ferreira et al. (2015) to sulfisoxazole and trimethoprim–sulfamethoxazole. In this study, *P. multocida* isolates were obtained from healthy cats. It is crucial to understand that phenotypic antimicrobial resistance alone is not a significant threat. The microbiota of cats consists of both pathogenic and non‐pathogenic bacteria. The antibiotic resistome (collection of all antibiotic resistance genes present in a microbial population or environment) in the human gut may be influenced by pet cats. Therefore, owners of companion animals should be mindful of the risk of resistome transmission. It is also important to take measures to minimize the transmission pathways of resistance derived from companion animals by managing their living environment and ensuring their health (Caballero‐Flores et al. 2023; Yang et al., 2023).

## 5 CONCLUSIONS

Companion animals, including cats and dogs, are known to be common reservoirs of *P. multocida* and can potentially transmit it to humans (Wilson & Ho, 2013). Research has indicated that human infection with *P. multocida* can occur through contact with dogs and cats, such as through biting, scratching or licking (Peng et al., 2022). This highlights the significant public health risk posed by *P. multocida* in humans. The epidemiological and molecular data from this study suggest that cats can harbor *P. multocida* in their oral cavity. The isolated organism from companion cats has been found to possess various virulence‐associated genes and exhibit resistance to certain antimicrobial drugs commonly used in both humans and animals. Additionally, capsular, LPS and virulotyping are valuable tools in genotyping *P. multocida* and serve as the foundation for designing region‐specific vaccines for future use. These approaches aid in understanding the pathogenicity of *P. multocida* and contribute to the development of effective preventive measures. Consequently, it is crucial not to overlook future public health concerns related to *P. multocida* in humans.

## AUTHOR CONTRIBUTIONS

*Supervision; conceptualization; methodology; formal analysis; writing – original draft; writing – review and editing*: Darioush Gharibi. *Supervision; methodology; investigation*: Bahman Mosallanejad. *Methodology*: Reza Avizeh. *Methodology; investigation*: Ali Ziagham. All authors read and approved the final manuscript.

## CONFLICT OF INTEREST STATEMENT

The authors declare that they have no known financial conflicts of interest or personal relationships that could have appeared to influence the work reported in this paper.

## FUNDING INFORMATION

Shahid Chamran University of Ahvaz, Khuzestan province, Iran, under grant number: SCU.VP98.261.

## ETHICS STATEMENT

The ethical approval was not required for the present study since the samples were collected from the mouth of cats.

## Data Availability

Data are available upon reasonable request to the corresponding author.
